# Selections

**Published:** 1892-03

**Authors:** 


### Selections.

### RUDOLPH VIRCHOW.

Rudolph Virchow, whose seventieth birthday was recently celebrated throughout the civilized world, justly ranks as the foremost pathologist of this century. Before the appearance of his work on "Cellular Pathology" our conception of pathological processes was based for the most part upon philosophical speculations. As one of his pupils, Professor Klebs, has recently said, it was Virchow who taught us to think microscopically, to read in the book of Nature, and recognize the minute pathological changes which had escaped the scrutiny of his predecessors. Virchow's axiom was *omnis cellula e cellula*, or as he says in his "Cellular Pathology," the cell is really the ultimate morphological element in which there is any manifestation of life. The demonstration of these views in his inimitable lectures effected as complete a revolution in medical thought as the French Revolution did in political economy. The development of bacteriology and subsequent investigations on cell life have served to modify some of Virchow's views, without, however, detracting in the least from their value. In his work on "Morbid Growths" he laid the foundation of our knowledge of neoplastic formations, and his numerous contributions to the pathology of various diseases, which are scattered through the *Archive of Pathological Anatomy* and other publications, have shed light on many subjects which previously were merged in obscurity. His writings on public hygiene, epidemiology, and military medicine have played an important part in effecting sanitary reforms. Although, strictly speaking, a pathologist, and always ready to uphold the independence of pathological anatomy, Virchow has never failed to recognize the claims of the clinician, and it is to him that we owe the intimate relation which pathology now bears to clinical medicine. Before his time there were great clinicians, but not scientific physicians in the true sense of the word, and many of the great medical authorities of the present day were trained under his guidance. —*Editorial in the International Journal of Surgery.*

